# Rapid Death from Oxalic Acid

**Published:** 1846-02

**Authors:** 


*Rapid Death from Oxalic Acid.*—Dr. Ogilvy, of Coventry, has communicated to the Lancet a case of poisoning by oxalic acid, in which it is probable that death took place within *three minutes* after the poison had been swallowed. The sister of the deceased had been absent from the room about that period, and on her return found her dying. The quantity of poison taken could not be determined. The only other remarkable circumstance in the case was, that the coats of the stomach were so softened, that on an attempt being made to remove the organ, they were lacerated by the weight of the contents. This was probably a chemical action post-mortem. The intestines and left lobe of the liver were also found softened, as if by transudation. We believe this to be the most rapidly fatal case on record.—*N. Y. Jour. of Med.*

